# The Physiology of the Carbohydrates: Their Application as Food and Relation to Diabetes

**Published:** 1894-12

**Authors:** 


*The Physiology of the Carbohydrates: their Application as Food and Relation to Diabetes.* By F. W. PAVY, M.D., F.R.S. Pp. x., 280. London: J. & A. CHURCHILL. 1894.

"A life's labour, attended with unceasing laboratory work, has been devoted to the attainment of the knowledge that has been acquired." This volume contains the record of what has been done, and the result is that the writer endeavours to overthrow the glycogenic doctrine which has conducted the minds of physiolgists in the wrong direction, and hence their search for knowledge has only resulted in fruitless gropings in the dark. "Through the recognition of the glucoside constitution of proteid matter, a clue was given which has led to the discovery of what I venture to regard as the true key to the situation." . . . "The liver, in fact, instead of doing what is claimed for it under the glycogenic doctrine, does exactly the reverse. It neither forms sugar to be discharged into the general circulation and conveyed to the tissues, nor temporarily stores up carbo-hydrate matter from ingestion, to be subsequently allowed as such to pass on. On the contrary, it keeps the general circulation free from the sugar that would otherwise enter and show itself in the urine. Its office is an arresting one in relation to carbo-hydrate matter; and if it were not for the exercise of this office, we should all be in the same position as the diabetic."

What, then, becomes of the carbo-hydrate matter? This question Dr. Pavy claims to have materially advanced in this research. He points out that the glycogenic doctrine throws no light upon this matter, inasmuch as it fails to show in what manner the sugar is disposed of in the tissues. Dr. Pavy deals with the disposal of carbo-hydrate matter by protoplasmic action under the respective heads: 1, Transmutation; 2, Application to the production of proteid; and 3, Transformation into fat. By these means, or one or other of them, carbo-hydrates of the food are disposed of in a certain manner, and if not so disposed of we have the condition known as diabetes.

Many of the points raised are such as will receive much criticism from physiologists and chemists; but the author speaks with authority on these matters, and his conclusions are worthy of the most careful consideration. He opens up a new field for the use of carbo-hydrate food when he concludes that the major part of the absorbed sugar is immediately combined with peptone to form a proteid, and he upsets some of the most cherished convictions of the older physiologists. There is much in the book which is new, and withal it is the outcome of steady, patient investigation; it is the work of a master in his subject, and he throws down the gauntlet with vigour and decision.

We shall await the details of the combat with much interest.

